# Catastrophic Esophageal Tumor Perforation within One Week after First-Line Chemo-Immunotherapy for Advanced Esophageal Squamous Cell Carcinoma: A Case Report

**DOI:** 10.70352/scrj.cr.25-0799

**Published:** 2026-03-06

**Authors:** Takeshi Matsubara, Shunsuke Kaji, Hiroki Okamura, Keisuke Inoue, Ayana Kishimoto, Kazunari Ishitobi, Takahito Taniura, Takayuki Tanaka, Tetsu Yamamoto, Masaaki Hidaka

**Affiliations:** Department of Digestive and General Surgery, Shimane University, Izumo, Shimane, Japan

**Keywords:** esophageal squamous cell carcinoma, esophageal perforation, chemo-immunotherapy, self-expandable metallic stent, source control

## Abstract

**INTRODUCTION:**

Esophageal perforation secondary to advanced esophageal squamous cell carcinoma (ESCC) is a life-threatening oncologic emergency that can rapidly progress to mediastinitis, empyema, and septic shock. With the increasing use of first-line chemo-immunotherapy, early tumor necrosis—particularly in ulcerated or deeply invasive lesions—may precipitate catastrophic perforation.

**CASE PRESENTATION:**

A man in his 70s was diagnosed with unresectable middle thoracic ESCC with distant metastases to the iliopsoas muscle and left supraclavicular lymph node (cT3brN2M1b, cStage IVB). First-line cisplatin plus 5-fluorouracil combined with pembrolizumab was initiated. On day 6 of cycle 1, he developed sudden, severe chest pain. CT revealed pneumomediastinum extending to the anterior mediastinum with periesophageal fluid collection; pleural effusion was minimal at presentation. Esophageal tumor perforation with mediastinitis was diagnosed, and broad-spectrum antibiotics and ventilatory support were started. Emergent endoscopy confirmed the perforation, and a covered self-expandable metallic stent was deployed. Right pleural drainage yielded grossly contaminated effusion, and the patient deteriorated to septic shock. In the ICU, aggressive source control was pursued with 3 pleural lavage and drainage procedures within the first 2 weeks (1 thoracoscopic and 2 open thoracotomy procedures). Although sepsis was controlled and his general condition temporarily improved after multimodal source control, systemic anticancer therapy could not be resumed. This clinical course underscores that even an ultra-early perforation during first-line chemo-immunotherapy can critically disrupt subsequent oncologic management, highlighting the need for rapid diagnosis, aggressive source control, and early goals-of-care discussions in unresectable disease.

**CONCLUSIONS:**

Tumor-related esophageal perforation can occur as early as day 6 during first-line chemo-immunotherapy for unresectable ESCC and may rapidly progress to fulminant mediastinitis and empyema. Once perforation is suspected, prompt diagnosis and an integrated, multidisciplinary, multimodal source-control strategy—endoluminal sealing with a covered stent plus timely and adequate thoracic drainage/lavage—should be prioritized, underscoring the clinical significance of ultra-early onset during the initial treatment period in the context of prior reports.

## Abbreviations

CCRT
: concurrent chemoradiotherapy
ECLNIE
: extracapsular lymph node involvement of the esophagus
EP
: esophageal perforation
ESCC
: esophageal squamous cell carcinoma
EVT
: endoscopic vacuum therapy
HPD
: hyperprogressive disease
IMRT
: intensity-modulated radiotherapy
PPD
: post-perforation day
PSS
: Pittsburgh Severity Score
SEMS
: self-expandable metallic stent
TPN
: total parenteral nutrition

## INTRODUCTION

EP associated with esophageal cancer is a life-threatening emergency that can rapidly progress to mediastinitis, empyema, sepsis, and multi-organ failure. Because patients are often critically ill at presentation, prompt diagnosis and multidisciplinary management—centered on infection source control—are essential; however, treatment is frequently limited to palliative measures in a substantial proportion of cases.^1,2)^

In recent years, first-line chemotherapy combined with an immune checkpoint inhibitor (chemo-immunotherapy) has become widely adopted for unresectable advanced or recurrent esophageal cancer, and cisplatin plus 5-fluorouracil with pembrolizumab is positioned as a standard regimen.^3)^ Meanwhile, analyses of risk factors for treatment-related EP have progressed, particularly in the radiotherapy setting. In patients with ESCC receiving radiotherapy, esophageal wall thickening, a “niche sign” on pretreatment contrast esophagography, and ECLNIE have been reported as independent risk factors.^2)^ ECLNIE is defined on contrast-enhanced CT by irregular nodal margins, adjacent fascial thickening, and apparent invasion into the esophageal wall, reflecting marked esophageal wall fragility due to en bloc tumor–nodal involvement.^2)^

Although case reports have described successful infection control followed by definitive surgery or resumption of anticancer therapy after tumor perforation during induction chemotherapy, early EP during first-line chemo-immunotherapy has rarely been reported.^4)^ Here, we report a case of unresectable advanced ESCC complicated by tumor perforation with severe mediastinitis and empyema on day 6 of the first chemo-immunotherapy cycle. Covered SEMS placement and repeated pleural lavage/drainage achieved transient recovery from septic shock; however, the perforation event necessitated discontinuation of systemic therapy, underscoring the clinical challenge of maintaining oncologic control after successful rescue.

## CASE PRESENTATION

**Fig. 1:**
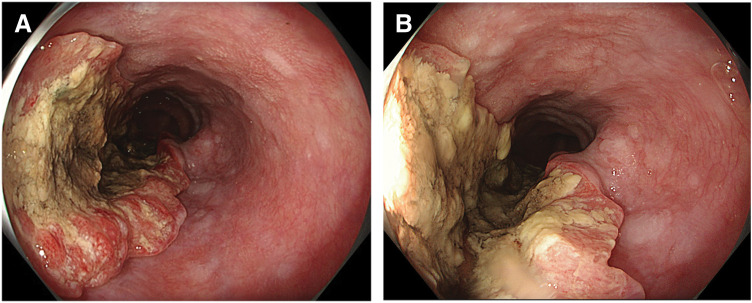
Baseline endoscopic findings. (**A**) Upper gastrointestinal endoscopy shows an advanced middle thoracic esophageal tumor with marked luminal narrowing and ulcerative changes. (**B**) Close-up view demonstrates deep ulceration with necrotic debris on the tumor surface.

A man in his 70s presented to a local hospital with progressive dysphagia and was referred to our institution for further evaluation and treatment. Upper gastrointestinal endoscopy revealed a tumor in the middle thoracic esophagus, and biopsy confirmed squamous cell carcinoma. Contrast-enhanced CT and PET-CT demonstrated metastases to the iliopsoas muscle and the left supraclavicular lymph node (station 104L). The disease was staged as unresectable ESCC (cT3brN2M1b, cStage IVB).^5,6)^ (**Figs. 1**–[Fig F2]**3**) Pretreatment contrast esophagography was not performed.

**Fig. 2 F2:**
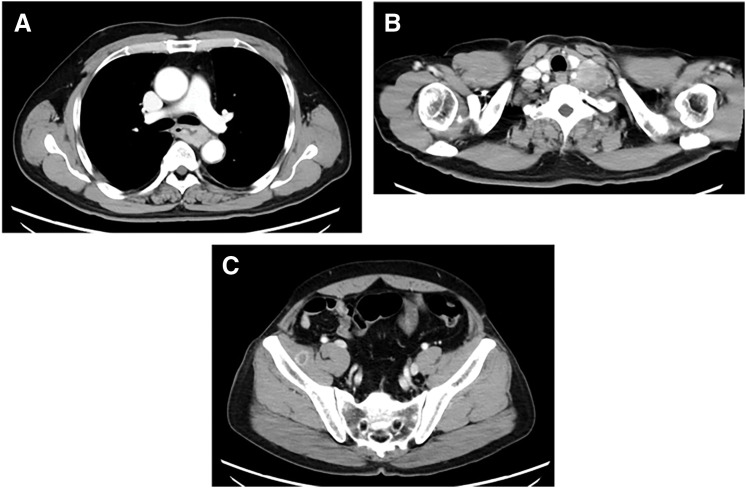
Staging contrast-enhanced CT at initial diagnosis. (**A**) Axial CT demonstrates an enhancing mass with marked circumferential wall thickening in the middle thoracic esophagus. (**B**) Axial CT shows left supraclavicular lymph node metastasis (station 104 L). (**C**) Axial CT demonstrates a metastatic lesion within the iliopsoas muscle.

**Fig. 3:**
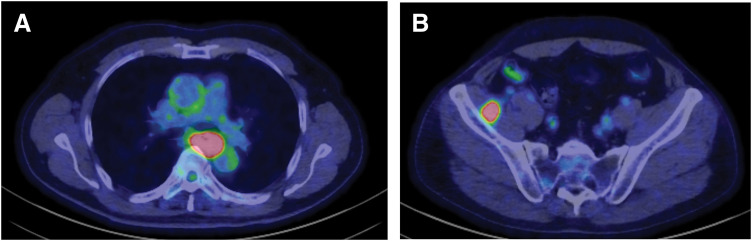
PET-CT at initial diagnosis. (**A**) PET-CT demonstrates FDG uptake in the primary esophageal lesion. (**B**) PET-CT shows intense FDG uptake in the iliopsoas muscle consistent with distant metastasis. FDG, fluorodeoxyglucose

First-line treatment was initiated according to current guidelines with cisplatin plus 5-fluorouracil combined with pembrolizumab. The regimen was as follows: pembrolizumab 200 mg on day 1; cisplatin 145 mg on day 1; and 5-fluorouracil 1450 mg as a continuous infusion on days 1–5. In this report, cycle day refers to days from initiation of cycle 1 chemo-immunotherapy (cycle day 1 = day of first drug administration). For the post-perforation course, we use PPD, where PPD0 corresponds to symptom onset on cycle day 6. During the initial days of treatment, no apparent adverse events were observed, and the patient reported improvement of dysphagia with adequate oral intake. This early symptomatic improvement suggested an early treatment response before the catastrophic event.

**Fig. 4:**
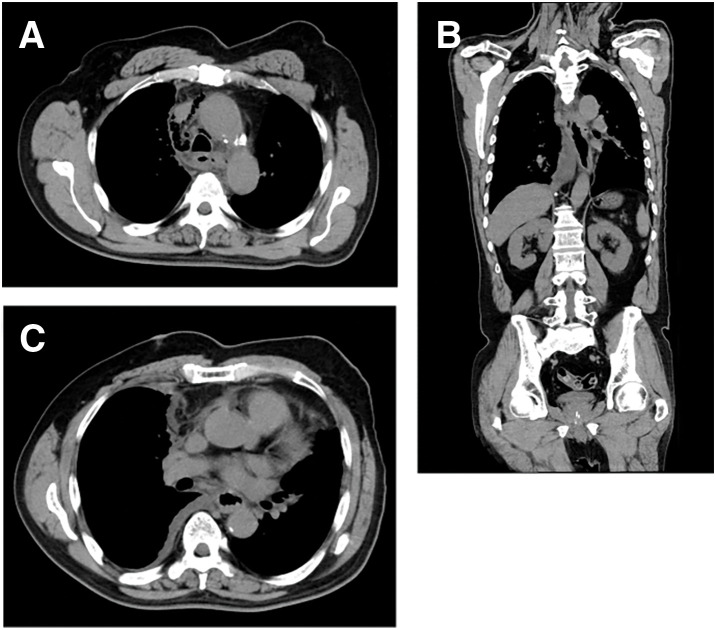
CT findings at the onset of perforation (day 6 of cycle 1). (**A**, **C**) Axial CT shows pneumomediastinum and periesophageal fluid collection compatible with esophageal perforation and mediastinitis. (**B**) Coronal CT demonstrates pneumomediastinum extending cranially toward the anterior mediastinum. Pleural effusion was minimal at this stage.

**Fig. 5:**
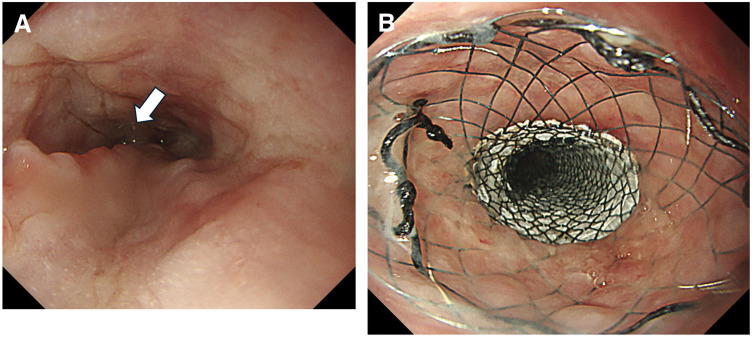
Emergent endoscopic management. (**A**) Endoscopy identifies the perforation site in the tumor (arrow). (**B**) A covered SEMS is deployed to cover the perforation (Niti-S flared covered stent, 18 × 150 mm). SEMS, self-expandable metallic stent

Late at night on day 6 of cycle 1(PPD0), he developed sudden, severe chest pain that was not relieved by analgesics. He had no preceding episodes of forceful vomiting or retching, and there was no recent endoscopic manipulation prior to symptom onset. Emergent CT revealed pneumomediastinum extending to the anterior mediastinum with periesophageal fluid collection (**Fig. 4**); right pleural effusion was minimal at that time. Based on these findings and the clinical course, tumor-related EP complicated by mediastinitis was diagnosed. Broad-spectrum antibiotics were initiated, and he required endotracheal intubation and mechanical ventilation. Because the event occurred on a holiday, emergent upper gastrointestinal endoscopy was performed approximately 5 h after the CT examination. Endoscopy identified the perforation site (**Fig 5A**, arrow), and a covered SEMS was deployed (Niti-S covered esophageal stent with flare; Taewoong Medical, Gimpo, Korea; 18 × 150 mm) (**Fig. 5B**). Simultaneous pleural drainage yielded grossly contaminated effusion from the right thoracic cavity, indicating pleural contamination secondary to mediastinal rupture. Although the primary tumor appeared predominantly on the left wall endoscopically, pleural contamination became right-sided. This laterality can be explained by mediastinal rupture with disruption of the mediastinal pleura and subsequent seeding into the right pleural cavity, which was also supported by intraoperative findings. He subsequently deteriorated to septic shock and was managed in the ICU with broad-spectrum antimicrobials, respiratory and circulatory support, and nutritional management. Long-term enteral access (e.g., feeding jejunostomy) was considered; however, it was deferred during the acute phase because the patient was in septic shock requiring intensive respiratory/circulatory support and repeated thoracic source-control procedures, and we aimed to avoid additional abdominal surgical burden. Nutritional support was therefore provided primarily via TPN.

At presentation, there was no clinically significant chemotherapy-related myelosuppression (WBC 6830/μL; neutrophils 5960/μL; platelets 267 × 10^3^/μL), suggesting that the fulminant sepsis was primarily driven by mediastinal/pleural contamination.

Blood cultures obtained on PPD1 were positive. Pleural fluid cultures from the right thoracic drainage grew *Streptococcus anginosus* and *Streptococcus constellatus*, and Gram staining demonstrated Gram-positive cocci. Follow-up cultures obtained after adequate source control were negative.

Empiric antimicrobial therapy was initiated immediately after diagnosis with meropenem (1 g every 12 h) plus vancomycin (dosed according to trough concentrations). Given the risk of contamination associated with EP, anaerobic coverage was considered sufficient with meropenem. Although pleural space infection/empyema developed, antifungal therapy was not added. After microbiological results became available, antimicrobial therapy was de-escalated to ampicillin on PPD5. Intravenous antimicrobial therapy was continued for a total of 60 days; thereafter, the regimen was discontinued/changed in response to recurrent pneumonia.

At the first operation (thoracoscopic pleural lavage and drainage), the right thoracic cavity showed massive contamination with turbid pleural effusion and diffuse pleural inflammation. The mediastinal pleura was ruptured, and contamination extended from the mediastinum into the pleural space, consistent with mediastinal rupture secondary to tumor perforation. Extensive irrigation with 10 L of warm saline was performed, loculations were broken down, and drains were placed near the diaphragm and the apex. During subsequent open thoracotomy lavage procedures, dense fibrin peel with copious purulent discharge was observed. The degree of pleural contamination was judged severe, and repeated washouts were required until the drainage became serous.

**Fig. 6:**
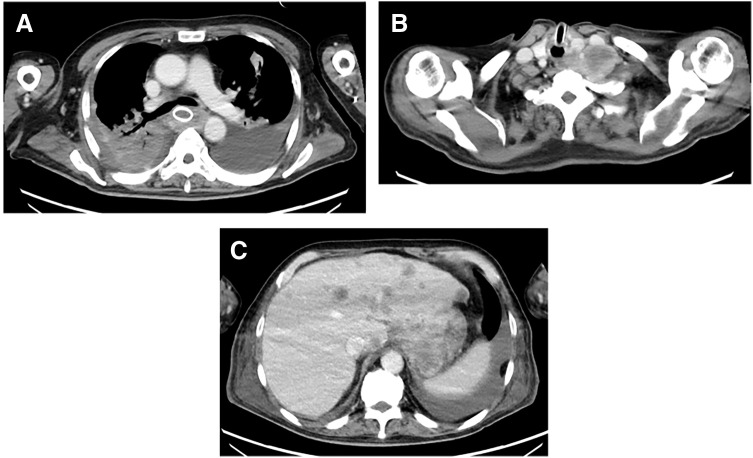
Follow-up CT demonstrating rapid disease progression. (**A**) Axial CT shows progressive left supraclavicular nodal disease. (**B**) Axial CT demonstrates bilateral pleural effusions/pleural disease after recovery from the acute septic episode. (**C**) Axial CT shows multiple liver metastases, consistent with rapid systemic progression.

**Fig. 7:**
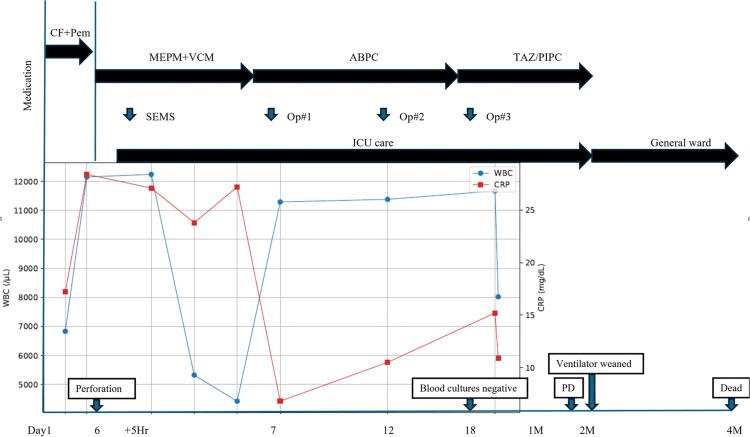
Clinical timeline and laboratory trends after tumor-related esophageal perforation. The upper panel summarizes the chronological clinical course from initiation of first-line chemo-immunotherapy, including major interventions and management (covered SEMS placement and thoracic lavage/drainage procedures) and antimicrobial therapy (MEPM+VCM, ABPC, and TAZ/PIPC, as shown). The lower panel shows serial changes in WBC (left y-axis,/μL) and CRP (right y-axis, mg/dL) over the same time axis. Key clinical milestones are indicated (perforation onset, blood cultures becoming negative, ventilator weaning, radiologic PD, and death). ABPC, ampicillin; CF, CDDP/5-FU; CRP, C-reactive protein; MEPM, meropenem; PD, progressive disease; Pem, pembrolizumab; SEMS, self-expandable metallic stent; TAZ/PIPC, piperacillin-tazobactam; VCM, vancomycin; WBC, white blood cell count

For definitive source control, pleural lavage and drainage were performed 3 times within 2 weeks after perforation onset: 1 thoracoscopic procedure and 2 open thoracotomy procedures. Thereafter, inflammatory markers and the patient’s general condition temporarily improved; he was weaned from septic shock and successfully liberated from mechanical ventilation. He became communicative and tolerated small amounts of oral intake. However, continuation of systemic anticancer therapy was not feasible after the perforation. Approximately 2 months after treatment initiation, follow-up imaging demonstrated rapid disease progression (**Fig. 6**). He ultimately died of respiratory failure 124 days after treatment initiation. The overall clinical course, including key diagnostic findings, major interventions (covered SEMS placement and pleural lavage/drainage on PPD2, PPD6, and PPD12), antimicrobial changes, and outcomes, is summarized in **Fig. 7**.

## DISCUSSION

This case is clinically notable because catastrophic esophageal tumor perforation occurred very early during first-line chemo-immunotherapy (day 6 of cycle 1) for unresectable ESCC, rapidly progressing to severe mediastinitis, empyema, and septic shock. EP associated with esophageal cancer is a life-threatening oncologic emergency that can lead to overwhelming infection and organ failure; outcomes are determined largely by prompt diagnosis and multidisciplinary management centered on infection source control.^1)^ In addition, treatment-related perforation may force discontinuation of systemic anticancer therapy and thereby profoundly affect subsequent oncologic outcomes. As chemo-immunotherapy is increasingly adopted as a standard first-line approach, reports of perforation occurring during the induction phase remain limited. In the KEYNOTE-590 publication, the incidence of EP was not explicitly described in the main text.^3)^ Accordingly, this case highlights a practical dilemma in the chemo-immunotherapy era: life-saving source control may be achievable, yet continuation of oncologic treatment can become infeasible. In the following sections, we discuss (i) high-risk features and potential mechanisms predisposing to perforation, (ii) therapeutic decision-making regarding stenting versus surgical options for leak control and infection management, and (iii) the challenge of resuming anticancer therapy after recovery, including how to interpret rapid deterioration without undermining the established concept of hyperprogressive disease.

Risk factor analyses of EP during radiotherapy/chemoradiotherapy provide a useful framework for understanding why catastrophic perforation can occur when a structurally vulnerable lesion is exposed to a potent antitumor effect. Chen et al. analyzed 306 patients with ESCC receiving radiotherapy and reported an EP incidence of 8.5%; older age, esophageal wall thickening, a “niche sign” on pretreatment contrast esophagography, and ECLNIE were identified as independent risk factors.^2)^ ECLNIE—defined on contrast-enhanced CT by irregular nodal margins, adjacent fascial thickening, and apparent invasion into the esophageal wall—likely represents en bloc tumor–nodal involvement with marked disruption and fragility of the esophageal wall. In this setting, a mismatch between rapid tumor regression/necrosis and the capacity for normal tissue repair has been proposed as a plausible mechanism underlying perforation.^2)^

We estimated the baseline risk in the present patient. Applying the published high-risk framework (wall thickening, niche sign, and ECLNIE), we considered this patient to be at elevated risk at baseline because pretreatment CT demonstrated marked circumferential esophageal wall thickening and endoscopy showed marked luminal narrowing with ulcerative changes, suggesting substantial wall compromise and stenosis. A pretreatment contrast esophagography was not performed, and therefore a “niche sign” could not be assessed. In addition, while lymph node metastasis was present, there were no clear pretreatment CT findings consistent with ECLNIE as defined by irregular nodal margins with apparent invasion into the esophageal wall. Overall, the combination of marked wall thickening and an ulcerated/stenotic lesion supported a high index of suspicion and the need for strict early monitoring.

This conceptual model—“a structurally fragile esophageal wall plus a potent treatment effect”—is supported by other radiotherapy-related reports. Pao et al. found that T4 disease and pretreatment esophageal stenosis were independent predictors of esophageal fistula/perforation after definitive CCRT with IMRT.^7)^ Consistently, a systematic review and meta-analysis identified ulcerative morphology, T4 stage, stenosis, and fluorouracil-based regimens among the factors associated with radiotherapy-related esophageal fistula/perforation.^8)^ Collectively, these findings suggest that deeply invasive, ulcerated, and/or stenotic tumors with substantial wall compromise are particularly susceptible to perforation when therapy induces rapid tumor necrosis and shrinkage.^2,7,8)^

Importantly, a similar mechanism may operate even without radiotherapy. Chemotherapy alone has been reported to precipitate perforation through abrupt tumor necrosis and regression, underscoring the need to recognize perforation as a potential complication of highly effective induction regimens.^4)^ Moreover, perforation during immune checkpoint inhibitor therapy has also been reported,^9)^ suggesting that, in the chemo-immunotherapy era, clinicians should not limit vigilance to radiotherapy settings alone. Therefore, before initiating first-line chemo-immunotherapy, careful review of baseline imaging for high-risk features—marked wall thickening, deep ulceration, tight stenosis, T4-equivalent invasion, or ECLNIE-like nodal invasion—may help identify patients at elevated risk. In such patients, strict early monitoring for new chest pain, fever, or respiratory compromise, along with a low threshold for repeat imaging, is warranted. In the present case, baseline endoscopy demonstrated deep ulceration with necrotic debris (**Fig. 1B**), suggesting a structurally vulnerable wall. Although the mucosal surface at emergency endoscopy appeared partially epithelialized (**Fig. 5A**), the CT findings and subsequent gross pleural contamination support rapid transmural tumor necrosis and wall breakdown, consistent with a “vulnerable wall plus abrupt (deep) necrosis” paradigm. Regarding laterality, air and infected fluid may initially accumulate within the mediastinum and subsequently enter the pleural space once the mediastinal pleura ruptures. In our case, CT initially showed pneumomediastinum with only minimal pleural effusion, followed by grossly contaminated right pleural effusion. Intraoperatively, the mediastinal pleura was ruptured and contamination extended from the mediastinum into the pleural space, supporting mediastinal-to-right-pleural spread as the mechanism of right-side dominance.

In our patient, dysphagia improved during the initial days of treatment with maintained oral intake, suggesting at least partial early tumor decompression. Prior studies propose that perforation can occur when a structurally fragile esophageal wall is exposed to a potent treatment effect, particularly through a mismatch between rapid tumor regression/necrosis and the capacity for normal tissue repair. Collectively, given the baseline ulcerated/compromised lesion and the extremely short interval to onset, we consider that an early and strong treatment response with rapid deep necrosis likely contributed to wall breakdown and perforation, even if the mucosal surface at emergent endoscopy appeared partially epithelialized. Although a sudden rise in intraluminal pressure (e.g., vomiting/retching) can precipitate esophageal rupture, no such precipitating event was evident/documented in our case. Given the baseline ulcerated and structurally fragile lesion and the extremely early timing after treatment initiation, we consider that rapid deep tumor necrosis and transmural wall breakdown was the primary mechanism, rather than pressure-related rupture.

In EP, particularly when associated with malignancy, outcomes are determined not by closure of the defect alone but by how rapidly and effectively infection source control is achieved. Contemporary reviews emphasize early, multidisciplinary management incorporating broad-spectrum antimicrobials, hemodynamic and respiratory support, nutritional management, and timely drainage of the mediastinum and/or pleural space, with endoscopic and surgical options selected according to anatomy and severity.^1,10)^ Severity stratification systems such as the PSS have been proposed to guide escalation of therapy, as more severe presentations often require more invasive interventions and carry a worse prognosis.^11)^ We retrospectively calculated the PSS at presentation. Based on the documented findings—malignancy (3 points), non-contained leak with mediastinitis/periesophageal fluid collection (2 points), respiratory compromise requiring mechanical ventilation (2 points), and pleural effusion (1 point)—the minimum PSS was 8, corresponding to the high-severity category (>5).

This high severity supports our strategy prioritizing prompt multimodal source control (covered stenting plus aggressive pleural/mediastinal drainage and lavage) rather than definitive oncologic surgery during the acute septic phase. To contextualize the clinical position of the present case, we summarized previously reported cases/series of esophageal cancer–related perforation, including triggers, timing, management strategies, and key outcomes (**Table 1**). This comparison highlights the exceptionally early onset in our patient (day 6 of cycle 1) and supports the importance of strict early monitoring and rapid multimodal source control.

**Table 1:** Literature summary of malignant esophageal perforation: setting/trigger, timing, management, and key outcomes (including the present case)

First author	Report type	Setting/trigger	Timing	Main management	Key outcome/implication
Ferri (2005)^12)^	Case report	Spontaneous perforation of advanced EC	NR	Covered SEMS	Stent-based management as a less-invasive option in malignant perforation
Kobayashi (2019)^4)^	Case report	Perforation during DCF chemotherapy	Day 4 of cycle 1	Drainage + stent → surgery	Staged strategy (source control → definitive management) feasible in selected case
Ohsawa (2020)^20)^	Case report	EC with perforation	NR	Esophageal bypass → definitive CRT	“Bridge” strategy enabling definitive therapy after stabilization
Schweigert (2016)^11)^	Retrospective series	Perforation after CRT for EC		Emergency esophagectomy	Rescue surgery experience in high-severity post-CRT setting
Griffiths (2009)^21)^	Retrospective series	Esophageal perforation (mixed; includes malignancy)	NR	Mixed	Prognostic context; malignant cases tend to be severe/poor outcomes
Chen (2014)^22)^	Cohort analysis	Perforation during/after RT for EC	NR	Mixed	Risk/prognostic factors for RT-associated perforation; informs risk discussion
Present case	Case report	Tumor-related perforation during first-line chemo-immunotherapy	Day 6 of cycle 1	Covered SEMS + repeated thoracic source control + TPN	Ultra-early onset; emphasizes strict early monitoring + rapid multimodal source control

CRT, chemoradiotherapy; DCF, docetaxel/cisplatin/5-fluorouracil; EC, esophageal cancer; NR, not reported; RT, radiotherapy; SEMS, self-expandable metallic stent; TPN, total parenteral nutrition

For immediate leak coverage, placement of a covered SEMS is an important minimally invasive option. Stent-based management for malignant EP has been described using covered SEMS.^12)^ A multi-institutional database analysis comparing surgical repair with stent-based management suggests that, with appropriate case selection and acknowledgment of selection bias, stenting can be an effective alternative strategy for perforation management.^13)^ However, SEMS primarily provides luminal diversion and leak occlusion and, by itself, does not eradicate established mediastinitis or empyema. Therefore, when a mediastinal abscess and/or pleural empyema is present, definitive treatment hinges on adequate drainage, lavage, and debridement as needed. In malignant perforation complicated by a mediastinal abscess, a combined approach using covered stenting and active drainage has been reported to achieve infection control and facilitate stabilization, while recognizing that tumor biology and disease stage frequently constrain the overall outcome.^14)^ In addition to SEMS, EVT has emerged as another endoluminal option for defect management. Accumulating evidence suggests that EVT may be considered depending on defect geometry and location and the risk of stent migration, typically as part of an integrated source-control strategy rather than a standalone solution.^15)^

In the present case, unresectable cStage IVB disease, septic shock, and grossly contaminated right pleural effusion indicated a fulminant infectious process with extensive thoracic contamination. Under these circumstances, emergent radical surgery aimed at definitive oncologic resection or complex esophageal reconstruction would be unlikely to provide meaningful oncologic benefit and would entail prohibitive physiological stress. Instead, the therapeutic priority appropriately shifted to life-saving source control: covered SEMS placement for leak coverage combined with aggressive pleural drainage and lavage. In this case, this approach required 3 interventions within 2 weeks (1 thoracoscopic and 2 open thoracotomy procedures). This strategy is consistent with contemporary principles emphasizing that endoscopic versus surgical interventions should be tailored to the severity of illness and contamination burden, prioritizing rapid infection control over definitive defect closure alone in critically ill patients. A more definitive diversion procedure (e.g., esophagectomy with cervical esophagostomy and feeding jejunostomy) was considered during multidisciplinary discussions. However, at the time of the first and second interventions, the patient was in the acute phase of septic shock with extensive mediastinal/pleural contamination, and additional major resection/diversion was judged to carry prohibitive physiological risk. Moreover, because the disease was unresectable with distant metastasis at baseline, the potential oncologic benefit of salvage resection was limited. Therefore, we prioritized a staged strategy focused on prompt multimodal source control (covered stenting plus repeated pleural/mediastinal washout and drainage), with the intent to preserve the possibility of resuming systemic therapy after stabilization.

Although multimodal source control achieved transient recovery from septic shock in this case, the perforation event effectively terminated systemic anticancer therapy, and the patient died of rapid disease progression approximately 4 months after treatment initiation. This trajectory highlights a critical feature of malignant EP in the chemo-immunotherapy era: even when the acute septic episode is survivable, the subsequent oncologic course may be dictated by the inability to promptly resume effective systemic therapy. Reviews emphasize that, particularly in malignancy-associated perforation, post-rescue decision-making should integrate overall disease status, physiological reserve, and goals of care, because the underlying cancer often becomes the principal determinant of survival once the infectious crisis is controlled.^10)^

HPD has been described after immune checkpoint inhibitor therapy in some malignancies, including esophageal cancer, and has also been reported across several solid tumors treated with programmed cell death protein-1/programmed death-ligand 1 blockade.^16–18)^ However, establishing HPD requires standardized baseline and on-treatment imaging with an appropriate time interval and quantitative assessment of tumor growth kinetics, and definitions/diagnostic thresholds remain heterogeneous.^18,19)^ In the present case, because systemic anticancer therapy was discontinued immediately after perforation and the imaging schedule was constrained by the septic course, the necessary conditions for a formal HPD evaluation were not met; therefore, we cannot conclude that this case represents HPD.

Accordingly, the key lesson is twofold. First, in patients with high-risk local features (marked wall thickening, ulceration, tight stenosis, and/or nodal invasion patterns suggesting wall fragility), clinicians should maintain heightened vigilance during the induction phase of chemo-immunotherapy, recognizing that catastrophic perforation can abruptly shift priorities to life-saving source control. Second, in unresectable advanced disease, early discussions with the patient and family regarding the treatment plan and priorities of care are essential once perforation occurs, because even successful rescue may preclude timely resumption of anticancer therapy and thereby limit meaningful oncologic benefit.

## CONCLUSIONS

We report a case of unresectable advanced ESCC complicated by catastrophic tumor perforation on day 6 of first-line chemo-immunotherapy, leading to severe mediastinitis, empyema, and septic shock. Although covered SEMS placement and repeated pleural lavage/drainage achieved temporary sepsis control, the perforation event precluded timely resumption of systemic anticancer therapy and was followed by rapid loss of oncologic control. In the chemo-immunotherapy era, careful pre-treatment assessment of high-risk local features and vigilant early monitoring are essential.

## DECLARATIONS

### Availability of data and materials

All data generated or analyzed during this study are included in this published article.

## References

[1] Nachira D, Sassorossi C, Petracca-Ciavarella L, et al. Management of esophageal perforations and postoperative leaks. Ann Esophagus 2023; 6: 10.

[2] Chen C, Fu X, Dai Y, et al. Impact of extracapsular lymph node involving the esophagus in esophageal perforation during and after radiotherapy: a propensity score-matched analysis. Cancer Manag Res 2020; 12: 6541–51. 32801892 10.2147/CMAR.S265273 PMC7398679

[3] Sun JM, Shen L, Shah MA, et al. Pembrolizumab plus chemotherapy versus chemotherapy alone for first-line treatment of advanced oesophageal cancer (KEYNOTE-590): a randomised, placebo-controlled, phase 3 study. Lancet 2021; 398: 759–71. 34454674 10.1016/S0140-6736(21)01234-4

[4] Kobayashi T, Makino T, Yamasaki M, et al. Successful stenting followed by surgery for perforated esophageal cancer due to chemotherapy. Ann Thorac Surg 2019; 108: e361–3. 31102633 10.1016/j.athoracsur.2019.04.022

[5] Mine S, Tanaka K, Kawachi H, et al. Japanese Classification of Esophageal Cancer, 12th Edition: Part I. Esophagus 2024; 21: 179–215. 38568243 10.1007/s10388-024-01054-y PMC11199297

[6] Doki Y, Tanaka K, Kawachi H, et al. Japanese Classification of Esophageal Cancer, 12th Edition: Part II. Esophagus 2024; 21: 216–69. 38512393 10.1007/s10388-024-01048-w PMC11199314

[7] Pao TH, Chen YY, Chang WL, et al. Esophageal fistula after definitive concurrent chemotherapy and intensity modulated radiotherapy for esophageal squamous cell carcinoma. PLoS One 2021; 16: e0251811. 33989365 10.1371/journal.pone.0251811 PMC8121322

[8] Zhu C, Wang S, You Y, et al. Risk factors for esophageal fistula in esophageal cancer patients treated with radiotherapy: a systematic review and meta-analysis. Oncol Res Treat 2020; 43: 34–41. 31639800 10.1159/000503754

[9] Shimomatsuya T, Hashimoto K, Nakamura S, et al. A case of perforation of unresectable esophageal cancer during nivolumab therapy (in Japanese). Gan To Kagaku Ryoho 2022; 49: 1105–7. 36281603

[10] Shaqran TM, Engineer R, Abdalla EM, et al. The management of esophageal perforation: a systematic review. Cureus 2024; 16: e63651. 39092389 10.7759/cureus.63651 PMC11293018

[11] Schweigert M, Sousa HS, Solymosi N, et al. Spotlight on esophageal perforation: a multinational study using the Pittsburgh esophageal perforation severity scoring system. J Thorac Cardiovasc Surg 2016; 151: 1002–11. 26897241 10.1016/j.jtcvs.2015.11.055

[12] Ferri L, Lee JK-T, Law S, et al. Management of spontaneous perforation of esophageal cancer with covered self expanding metallic stents. Dis Esophagus 2005; 18: 67–9. 15773847 10.1111/j.1442-2050.2005.00453.x

[13] Gray KE, Sarode A, Jiang B, et al. Surgical repair vs stent for esophageal perforation: a multi-institutional database analysis. Ann Thorac Surg 2023; 115: 1378–84. 35921860 10.1016/j.athoracsur.2022.07.023

[14] Han X, Zhao YS, Fang Y, et al. Placement of transnasal drainage catheter and covered esophageal stent for the treatment of perforated esophageal carcinoma with mediastinal abscess. J Surg Oncol 2016; 114: 725–30. 27654983 10.1002/jso.24384

[15] Luttikhold J, Pattynama LMD, Seewald S, et al. Endoscopic vacuum therapy for esophageal perforation: a multicenter retrospective cohort study. Endoscopy 2023; 55: 859–64. 36828030 10.1055/a-2042-6707 PMC10465237

[16] Champiat S, Dercle L, Ammari S, et al. Hyperprogressive disease is a new pattern of progression in cancer patients treated by anti-PD-1/PD-L1. Clin Cancer Res 2017; 23: 1920–8. 27827313 10.1158/1078-0432.CCR-16-1741

[17] Kim CG, Kim KH, Pyo KH, et al. Hyperprogressive disease during PD-1/PD-L1 blockade in patients with non-small-cell lung cancer. Ann Oncol 2019; 30: 1104–13. 30977778 10.1093/annonc/mdz123

[18] Champiat S, Ferrara R, Massard C, et al. Hyperprogressive disease: recognizing a novel pattern to improve patient management. Nat Rev Clin Oncol 2018; 15: 748–62. 30361681 10.1038/s41571-018-0111-2

[19] Ferrara R, Mezquita L, Texier M, et al. Hyperprogressive disease in patients with advanced non-small cell lung cancer treated with PD-1/PD-L1 inhibitors or with single-agent chemotherapy. JAMA Oncol 2018; 4: 1543–52. 30193240 10.1001/jamaoncol.2018.3676 PMC6248085

[20] Ohsawa M, Hamai Y, Ibuki Y, et al. Successful management of esophageal cancer with perforation using bypass surgery followed by definitive chemoradiotherapy. In Vivo 2020; 34: 2169–72. 32606200 10.21873/invivo.12025 PMC7439868

[21] Griffiths EA, Yap N, Poulter J, et al. Thirty-four cases of esophageal perforation: the experience of a district general hospital in the UK. Dis Esophagus 2009; 22: 616–25. 19302220 10.1111/j.1442-2050.2009.00959.x

[22] Chen H, Ma X, Ye M, et al. Esophageal perforation during or after conformal radiotherapy for esophageal carcinoma. J Radiat Res 2014; 55: 940–7. 24914102 10.1093/jrr/rru031 PMC4202289

